# Low Hepatitis B Immunity Among Ukrainian Refugee Children and Adolescents in Poland: Need for Targeted Screening and Vaccination

**DOI:** 10.3390/vaccines13080816

**Published:** 2025-07-31

**Authors:** Lidia Stopyra, Karolina Banach, Magdalena Wood, Justyna Stala, Anna Merklinger-Gruchała

**Affiliations:** 1 Department of Infectious and Tropical Diseases, Andrzej Frycz Modrzewski Krakow University Medical College, 30-705 Kraków, Poland; 2 Department of Infectious Diseases and Pediatrics, Zeromski Specialist Hospital, 31-913 Kraków, Poland; 3 Department of Bioinformatics and Public Health, Andrzej Frycz Modrzewski Krakow University Medical College, 30-705 Kraków, Poland

**Keywords:** migrants, children, preventions, prevalences, antibodies, HBV immunity, vaccination coverage, Poland, serological testing, public health

## Abstract

Background: The 2022 conflict in Ukraine triggered mass migration, leading to a significant influx of Ukrainian refugee children into Poland. This situation raises concerns about hepatitis B virus immunity, as Ukraine’s hepatitis B vaccination coverage has been inconsistent compared to Poland’s high vaccination rates. Objective: To evaluate hepatitis B immunity and infection prevalence among Ukrainian refugee children residing in Southern Poland and to assess implications for vaccination strategies in the host country. Methods: A prospective cross-sectional study was conducted on 1322 Ukrainian refugee children (0–18 years) presenting to a pediatric infectious diseases department in Southern Poland between February 2022 and March 2024. Data on vaccination history, demographic characteristics, and selected laboratory parameters, including hepatitis B surface antigen and anti-HBs antibody levels, were collected. Protective immunity was defined as anti-HBs antibody levels ≥10 IU/L. Results: Among the participants (mean age 9.9 years; 50.2% female), 83.2% were reported as vaccinated according to national immunization programs, but only 64.9% demonstrated protective anti-HBs antibody levels. Protective antibody prevalence declined significantly with age, with less than half of adolescents aged 15–18 years showing immunity. Five children (0.4%) were diagnosed with chronic hepatitis B, four of whom were unvaccinated. Conclusions: This study identifies a significant gap in hepatitis B immunity among Ukrainian adolescent refugees residing in Southern Poland, with less than half possessing protective anti-HBs antibody levels. This immunity gap and the high risk of sexual transmission of the hepatitis B virus in adolescents highlight the urgent need for comprehensive surveillance, screening, and catch-up vaccination programs.

## 1. Introduction

The outbreak of war in Ukraine on 24 February 2022 triggered one of the largest migration crises in Europe in recent decades. Since that time, over 12 million individuals have crossed the Polish–Ukrainian border, with approximately 6 million Ukrainian refugees officially registered across Europe as of 19 August 2024, according to data from the United Nations High Commissioner for Refugees (UNHCR). In the Małopolska region of Poland alone, 53,921 refugees from Ukraine have been recorded, the majority being women and children [1].

Migration on such a large scale inevitably leads to significant epidemiological consequences for host countries. It can impact public health infrastructure, affect immunization programs, and alter the prevalence and transmission dynamics of infectious diseases. Among the key public health priorities during humanitarian crises is the verification and reinforcement of immunization coverage, especially for vaccine-preventable diseases with serious clinical outcomes, such as hepatitis B.

Before the full-scale Russian invasion, in 2020, the estimated prevalence of hepatitis B surface antigen (HBsAg) among Ukrainian children under five years of age was 0.25% [0.18–0.34%] [2,3]. In certain regions, such as Zakarpattia in western Ukraine, serological studies conducted in 2017 found chronic hepatitis B virus (HBV) infection in 0.7% of children aged 2–11 years [4]. This situation likely resulted from insufficient vaccination coverage among children in Ukraine. Although the Ukrainian National Immunization Program (NIP) mirrors Poland’s schedule with a 3-dose hepatitis B vaccine series administered within the first seven months of life, the actual coverage has shown significant fluctuations over the years. After initially achieving coverage rates of 92–98% between 2004 and 2007, vaccination uptake dropped dramatically to 21–48% during 2010–2016, only to partially recover in the years 2017–2021 to levels between 77% and 81%. It is worth noting that the coronavirus disease 2019 (COVID-19) pandemic had a substantial impact on hepatitis B vaccination coverage. According to the World Health Organization/United Nations International Children’s Emergency Fund (WHO/UNICEF) data, coverage declined from 81% in 2020 to 77% in 2021, and further dropped to just 66% in 2022, following the outbreak of the war in Ukraine [2,5].

Such variability raises concerns about the consistency and completeness of immunization coverage among Ukrainian children.

In contrast, Poland has maintained high and consistent hepatitis B vaccination coverage since the nationwide introduction of the vaccine between 1994 and 1996, resulting in the near elimination of HBV infections among children by 2014 [6,7]. Between 2002 and 2012, the national coverage remained at a level of 97–98%. However, a significant decline has been observed in recent years, with the coverage rate decreasing to 89.3% in 2021. In 2022, the previously observed decline in vaccination coverage in 1-year-olds ended and the childhood vaccination rate increased to 89.8%, which is significant in the context of the goals set by the WHO hepatitis B elimination strategy [8].

The recent large-scale influx of child refugees—many of whom are unvaccinated or have incomplete immunization histories—may pose a significant challenge to the country’s previously stable epidemiological situation regarding hepatitis B.

WHO’s global hepatitis elimination strategy aims to eliminate viral hepatitis by 2030 [9]. The elimination is defined as a 90% reduction in new infections and a 65% reduction in mortality compared to 2015 levels. Pediatric-specific goals include ≥90% coverage with the three-dose hepatitis B vaccine series and reducing HBsAg prevalence in children under five to below 0.1% [10,11]. Every effort must be made to meet these targets, especially in the context of large-scale migration.

This study aimed to estimate the level of immunity against HBV and to evaluate the potential occurrence of HBV infections in a cohort of Ukrainian refugee children who were diagnosed and treated at a pediatric infectious diseases department in the southern region of Poland.

## 2. Materials and Methods

### 2.1. Study Design and Participants

This prospective cross-sectional study was conducted at a single pediatric infectious disease department. The study population comprised 1322 children and adolescents who underwent diagnostic and/or therapeutic evaluation between February 2022 and March 2024. The inclusion criteria were aged 0–18 years, had Ukrainian citizenship, and refugee status.

Data collection focused on demographic variables, vaccination status, presence of chronic diseases, and selected laboratory parameters, including the following: HBsAg, Anti-HBs, Anti-HBc (hepatitis B core antigen), Anti-HCV (hepatitis C antigen), and Anti-HIV (human immunodeficiency virus) antibodies, complete blood count (CBC) with differential, serum iron levels, vitamin D3 (25-OH metabolite), total IgE antibodies, and thyroid-stimulating hormone (TSH).

HBsAg, anti-HBs, anti-HBc, anti-HCV, and anti-HIV antibodies were analyzed using the Abbott Chemiluminescent Microparticle Immunoassay (CMIA).

Immunization histories were primarily obtained from medical records. In cases where documentation was missing—understandable in the context of fleeing a war zone—data were verified through parental recall.

### 2.2. Data Collection and Serological Testing

Anti-HBs antibody concentrations (IU/L) were measured to assess post-vaccination immunity. A concentration of ≥10 IU/L was defined as seroprotective, based on established guidelines [12,13,14]. Vaccination status was classified into four categories: fully vaccinated per NIP (at least 3 doses of the vaccine), unvaccinated, incompletely vaccinated (not completing all 3 doses of the vaccine), and unknown/missing data.

### 2.3. Statistical Analysis

Continuous variables were presented as means and standard deviations (SD) and medians with interquartile ranges (Q1–Q3). Group comparisons were conducted using the Mann–Whitney U test for two groups or the Kruskal–Wallis test for more than two groups. In cases of statistical significance, post hoc pairwise comparisons were performed.

Categorical variables were expressed as counts and percentages. Group differences were assessed using Pearson’s χ^2^ test. Where significant associations were found, post hoc analysis with standardized residuals was conducted to identify specific group differences. To assess age-related trends in the percentage of children with anti-HBs ≥10 IU/L and those reportedly vaccinated according to the National Immunization Program, the Mantel–Haenszel χ^2^ test was used.

All statistical analyses were performed using Statistica version 13.3 (StatSoft Inc., Tulsa, OK, USA) and jamovi software version 2.6.26. A *p*-value of less than 0.05 was considered statistically significant.

### 2.4. Ethical Approval

The study was performed in accordance with the ethical standards of the Declaration of Helsinki and its later amendments. Ethical approval was granted by the Bioethics Committee of the Regional Medical Chamber in Krakow, Nr 141/KBL/OIL/2022, on 28 November 2022. Informed consent was obtained from all participants or their legal guardians prior to inclusion in the study.

## 3. Results

### 3.1. Descriptive Characteristics of the Study Group (Table 1)

The descriptive characteristics of the study population are presented in Table 1.

A total of 1322 children met the inclusion criteria for the study (mean age 9.94 ± 4.49 years; 50.2% female). The median Body Mass Index (BMI) was 16.78 (IQR: 14.87–19.50). Vaccination status was documented for 99.8% of participants; however, only 5.2% had physically available vaccination records, whereas 94.8% relied on caregiver-reported histories.

Of the participants, 1100 (83.21%) children were declared vaccinated according to the NIP, 40 (3.0%) were unvaccinated, and 179 (13.5%) were incompletely vaccinated. Anti-HBs antibodies were detected in 75.6% of participants, but only 64.9% achieved seroprotective levels ≥ 10 IU/L.

A significant difference in anti-HBs antibody levels across vaccination groups was found (Kruskal–Wallis H (2, N = 1062) = 30.98; *p* < 0.001). Notably, among children declared fully vaccinated (per NIP), only 67.6% achieved seroprotective levels of antibodies. No significant difference in anti-HBs antibody levels was found between fully and incompletely vaccinated children (*p* = 0.93).

### 3.2. Comparison of Clinical and Laboratory Variables Stratified by Anti-HBs Serostatus (≥10 IU/L) (Table 2 and Table 3)

The comparison of Clinical and Laboratory data stratified by anti-HBs antibody status is presented in Table 2 and Table 3.

Children with anti-HBs antibody levels ≥10 IU/L were significantly older and had a higher BMI. The seropositive group also demonstrated significantly lower leukocyte and lymphocyte counts compared to seronegative participants.

Statistically significant associations were observed between anti-HBs serostatus and both eosinophil counts (*p* = 0.027) and serum TSH levels (*p* < 0.001). Children with eosinophilia or abnormal TSH levels were more likely to have anti-HBs antibody levels below the protective threshold.

Children with chronic diseases were less likely to achieve seroprotective anti-HBs levels (≥10 IU/L) (61.1% vs. 66.8%, *p* = 0.04).

Children whose chronic illness was diagnosed only following their arrival in Poland and who were subsequently referred for hospitalization or specialist care showed no significant difference in anti-HBs antibody levels compared to healthy children.

No significant differences were observed in serum iron, total IgE levels, or referrals to specialists or hospitals.

### 3.3. Relationship Between Chronic Illness and Vaccination Status (Table 4)

The comparison of vaccination status depending on the presence of major chronic diseases and referrals to specialist clinics and hospitals is presented in Table 4.

Laboratory and clinical analyses revealed that children with chronic illnesses were more frequently unvaccinated compared to those without chronic conditions (6.4% vs. 1.5%, *p* < 0.001). None of these children had documented contraindications for vaccination; however, incomplete immunization schedules in this group stemmed from illness-related delays or discontinuation of vaccines post-diagnosis.

### 3.4. Age-Related Trends in Antibody Positivity and Vaccination

**Figure 1:**
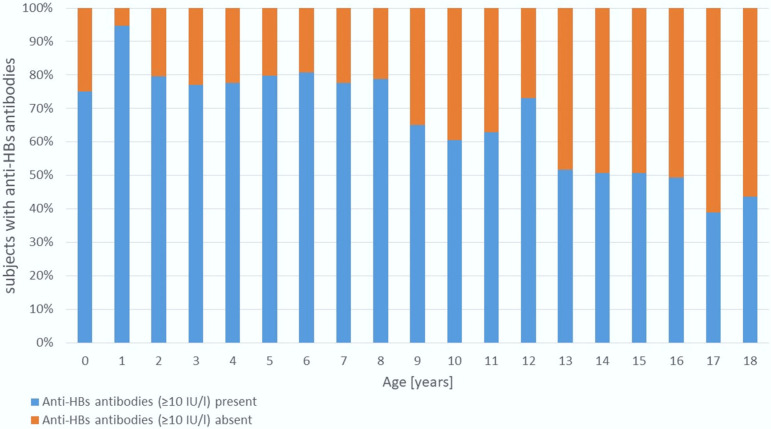
Age-Related Trends in Antibody Positivity.

**Figure 2:**
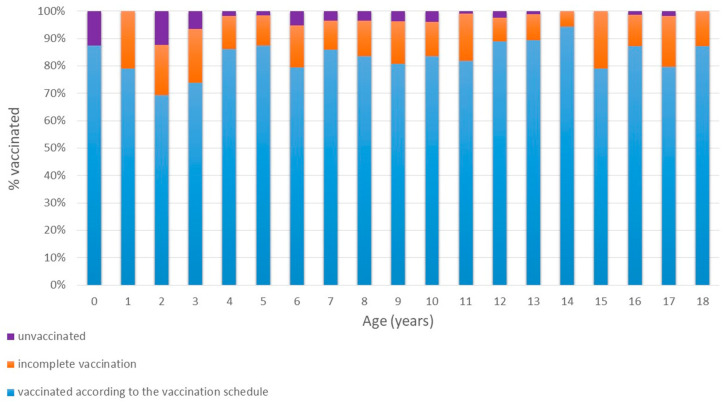
Age-Related Trends in Vaccination.

Two opposing but complementary trends were observed as follows (Figure 1 and Figure 2):

The proportion of children with seroprotective anti-HBs levels (≥10 IU/L) declined significantly with age (Mantel–Haenszel χ^2^ = 84.3, *p* < 0.001). Seropositivity exceeded 75% in children aged 1–8 years but decreased to 39–50% in adolescents aged 15–18 years.

In contrast, the rate of children declared as fully vaccinated (according to the NIP) increased with age (Mantel–Haenszel χ^2^ = 4.8; *p* = 0.028). The lowest declared full vaccination rate (69.4%) occurred among 2-year-olds, peaking at 94.4% in 14-year-olds.

### 3.5. Chronic Hepatitis B Cases

The most serious clinical outcomes such as liver damage, cirrhosis, liver cancer, and even death, are mostly associated with chronic hepatitis, the risk of which is greatest among young children. Five children (0.4%) aged 7, 12, 12, 13, and 14 years were diagnosed with chronic hepatitis B. One patient presented with hepatic cirrhosis secondary to chronic hepatitis B. The remaining four had comorbid conditions, predominantly oncologic diagnoses such as acute lymphoblastic leukemia (ALL), neuroblastoma, nephroblastoma, and one child had diagnosed cerebral palsy (CP). Four were unvaccinated, and one had vaccinations discontinued following a neuroblastoma diagnosis. All started antiviral treatment, with the child having hepatic cirrhosis being referred for liver transplantation.

## 4. Discussion

The Russian aggression in Ukraine in early 2022 precipitated the large-scale displacement of Ukrainian refugees into Europe. Both epidemiological reports and randomized controlled trials have highlighted the public health challenges posed by population movements. A meta-analysis by Hobart et al., encompassing 23 studies on pediatric refugee health, identified elevated HBV susceptibility among displaced populations, particularly migrants from Africa and Eastern Europe, with high rates of both HBV exposure and infection [10]. Similarly, Virk et al., in a review of 39 studies across 24 countries, reported that full vaccination coverage among refugee children was only 21%. Factors associated with improved immunization rates included having more children, higher parental education, and better access to healthcare [11].

Public awareness regarding the epidemiological implications of migration has also grown. A recent study from Central Poland showed that, despite ongoing vaccine hesitancy, 10% of Polish parents decided to vaccinate their children due to the arrival of Ukrainian refugees [15].

Various and coordinated national and local public health initiatives have been implemented to address immunization gaps among displaced populations. WHO recommended that Governments in Europe should provide free and accessible hepatitis care, as well as vaccinations, to Ukrainian refugees. Hepatitis B vaccinations should be offered to children and adolescents with unknown vaccination status, known delayed status, or missing vaccines, as well as to others with risk factors who do not have official records or evidence of immunity [16]. It was highlighted that the epidemiological surveillance in European countries should have been intensified and constantly monitored [17]. The United Kingdom Health Security Agency issued detailed recommendations to ensure that refugees from Ukraine are provided with prioritized and comprehensive healthcare [18]. The European Centre for Disease Prevention and Control (ECDC) has developed operational guidance for the prevention and control of infectious diseases in the context of increased migrant/refugee flows linked to the Ukraine crisis [6]. The main communicable diseases and health threats that have been identified by the ECDC are vaccine-preventable diseases [19].

In our cohort of Ukrainian refugee children, significant deficiencies in hepatitis B immunity (anti-HBs ≥ 10 IU/L) were identified. A critical challenge was the scarcity of documented vaccination histories—only 5.4% of participants had verifiable records, and in most cases, healthcare providers had to rely on verbal reports from caregivers. While understandable given the acute displacement from conflict zones, this lack of documentation compromises data veracity. Compounding this issue, some children were accompanied by non-parental caregivers (e.g., relatives or guardians), further undermining the credibility of the vaccination history. In some instances, caregivers may have deliberately concealed their unvaccinated status due to fear of restricted access to healthcare or education services. This uncertainty necessitates cautious epidemiological interpretation and, in some cases, the implementation of presumptive vaccination strategies to ensure adequate protection against vaccine-preventable diseases such as hepatitis B.

Similar documentation issues have been reported in other studies. For example, Hobart et al. noted that in 58.1–79.3% of refugee children, vaccination records were unavailable, and even when present, they often conflicted with serological findings [10].

There were also studies showing vaccine hesitancy among Ukrainian refugees. The efforts made to ensure complete care and to promote vaccination among refugees, offering them a complete evaluation of the vaccination status and the possibility of being vaccinated for free, seemed to be insufficient to convince most refugees to get vaccinated [20].

In our study, 83.2% of caregivers reported full hepatitis B vaccination, contrasting with pre-war Ukrainian national data documenting 62–77% vaccination completeness [18]. Among those claiming full vaccination, only 67.6% exhibited seroprotective anti-HBs levels (≥10 IU/L). This diverges from the established evidence showing that over 96% of children vaccinated in the first year of life (as recommended in Ukraine’s NIP) typically develop protective antibody levels [21,22]. Since our cohort (excluding those with HBV infection) did not include children with underlying serious chronic conditions associated with immunologic non-response, the most plausible explanation for this discrepancy is that the reported vaccination histories were inaccurate.

Therefore, it is critical to remain vigilant and avoid basing diagnostic or therapeutic decisions solely on self-reported immunization status.

When analyzing age-related trends in hepatitis B immunity, we observed that children up to age 8 had anti-HBs positivity rates above 75%. However, a particularly alarming finding was that only 39–52% of adolescents aged 15–18 years had anti-HBs antibody levels ≥10 IU/L (Figure 1), despite reportedly high vaccination coverage (Figure 2). While a natural decline in antibody levels over time may partially explain this finding, it does not fully account for such low seroprotection in adolescents. A more likely explanation is that vaccination was either incomplete or not administered at all, despite caregiver claims. Unfortunately, to the best of our knowledge no detailed data exist on hepatitis B vaccine coverage by age among Ukrainian children.

The suboptimal anti-HBs seroprotection rates (≥10 IU/L) observed in adolescents aged 15–18 years raise particular concern, given their heightened HBV exposure risk during initiation of sexual activity, a key risk factor for HBV transmission. Similar conclusions were drawn by Le MH et al., who, in a cross-sectional study conducted between 1999 and 2016, investigated post-HBV vaccination immunity and observed a significant decline in immunity among US-born adolescents aged 14–18 years [23]. Although vaccinated individuals may retain long-term immune memory even when antibodies wane, in our cohort, we cannot be certain of the authenticity of the reported vaccination status.

To the best of our knowledge, this is the first study to assess anti-HBs levels in Ukrainian refugee children by age group, and our results clearly identify a high-risk subgroup requiring urgent attention and targeted public health interventions.

In our study group, five children (0.4%) were diagnosed with chronic HBV infection. In four of these cases, the infection was linked to frequent hospitalizations and blood transfusions due to chronic illnesses, raising concerns about infection control and transfusion safety in Ukrainian healthcare facilities. These findings align with existing epidemiological data: according to WHO and ECDC estimates, hepatitis B prevalence in Ukraine ranges from 1 to 1.3% in the general population and 0.25% among children, with rates reaching up to 0.7% in some regions of Western Ukraine, the highest in Europe [2,3,24].

In contrast, the incidence of hepatitis B among children in Poland is just 0.01% [2]. Given the high volume of incoming refugees and the background HBV seroprevalence, screening, diagnostic, and treatment protocols for hepatitis B should be urgently implemented not only for adults but also for children.

## 5. Limitations

This study has several limitations. First, the relatively small sample size may limit the generalizability of findings to the broader population of Ukrainian adolescents in Poland. Second, the lack of verified vaccination records restricted our ability to correlate serological status precisely with vaccination history. Parental reporting, although understandable under refugee circumstances, may be affected by recall bias. Third, the cross-sectional design provides a snapshot but cannot ascertain the duration of seroprotection or immune memory. Finally, although anti-HBs, HBsAg, and anti-HBc were assessed, other immune markers or functional immunity assessments, which could provide a more comprehensive understanding of protection, were not included.

## 6. Conclusions

This study identifies a significant gap in hepatitis B immunity among Ukrainian adolescent refugees residing in Southern Poland, with less than half possessing protective anti-HBs antibody levels. This immunity gap, coupled with known low vaccination coverage in Ukraine and the high risk of the sexual transmission of HBV in adolescents, highlights the urgent need for surveillance, comprehensive screening, and catch-up vaccination programs. Targeted interventions will help protect vulnerable populations and sustain Poland’s low HBV prevalence.

Given that 32.6% of patients who reported full hepatitis B vaccination did not have a protective level of antibodies, clinical decisions should not rely solely on self-reported vaccination history.

## Figures and Tables

**Table 1:** Characteristics of the Study Group.

Characteristic	Value
**Age (years)**	
N	1317
Mean (SD)	9.94 (4.49)
Median (Q1–Q3)	10.00 (7.00–13.00)
**BMI**	
N	1308
Mean (SD)	17.59 (3.71)
Median (Q1–Q3)	16.78 (14.87–19.50)
**Anti-HBs antibodies (IU/L)**	
N	1065
Mean (SD)	184.01 (314.13)
Median (Q1–Q3)	20.00 (2.00–191.00)
**Anti-HBs antibodies (presence)**	
0 (none)	318 (24.05%)
1 (present)	999 (75.57%)
No available data	5 (0.38%)
**Anti-HBs antibodies (presence ≥10 IU/L)**	
1 (none)	463 (35.02%)
0 (present)	859 (64.98%)
No available data	0 (0.0%)
**Sex**	
0 (Male)	657 (49.70%)
1 (Female)	664 (50.23%)
No available data	1 (0.08%)

**Table 2:** Clinical and Laboratory Characteristics of Patients Stratified by Anti-HBs Antibody Status (≥10 IU/L vs. <10 IU/L) (N = 1322).

Characteristic		Anti-HBs Antibodies		
		0 (Positive, ≥10 IU/L)	1 (None)	*p*-Value
		N = 859	N = 463	
Age (years) ^φ^				
	Mean (SD)	11.50 (4.28)	9.11 (4.39)	<0.001
	Median (Q1–Q3)	12.00 (9.00–15.00)	9.00 (6.00–12.00)	
BMI ^φ^				
	Mean (SD)	18.63 (4.13)	17.03 (3.34)	<0.001
	Median (Q1–Q3)	18.08 (15.62–20.82)	16.19 (14.67–18.79)	
Leukocytes (WBC) [×10^3^/µL] ^φ^				
	Mean (SD)	6.85 (2.23)	7.44 (2.72)	<0.001
	Median (Q1–Q3)	6.54 (5.60–7.90)	6.90 (5.60–8.60)	
Neutrophils (NEUT) [×10^3^/µL] ^φ^				
	Mean (SD)	3.46 (1.43)	3.66 (2.93)	0.289
	Median (Q1–Q3)	3.22 (2.57–4.06)	3.10 (2.31–4.16)	
Lymphocytes (LYMPH) [×10^3^/µL] ^φ^				
	Mean (SD)	2.53 (1.17)	3.34 (3.59)	<0.001
	Median (Q1–Q3)	2.48 (1.95–3.08)	2.76 (2.22–3.63)	
Eosinophils, n (%) ^χ^				0.027
	Normal (0)	744 (66.02%)	377 (33.45%)	
	Abnormal (1)	111 (58.12%)	80 (41.88%)	
	No data	4 (40.00%)	6 (60.00%)	
Serum Iron, n (%) ^χ^				0.350
	Normal (0)	676 (65.38%)	350 (33.85%)	
	Abnormal (1)	178 (62.90%)	105 (37.10%)	
	No data	4 (33.33%)	8 (66.67%)	
Total IgE, n (%) ^χ^				0.817
	Normal (0)	535 (65.32%)	284 (34.68%)	
	Abnormal (1)	317 (64.69%)	173 (35.31%)	
	No data	7 (53.85%)	6 (46.15%)	
TSH, n (%) ^χ^				<0.001
	Normal (0)	813 (66.37%)	412 (33.63%)	
	Abnormal (1)	41 (47.67%)	45 (52.33%)	
	No data	5 (45.45%)	6 (54.55%)	

Legend: ^φ^ Mann–Whitney U test; ^χ^ Pearson Chi-square test; analyses were performed excluding unavailable data.

**Table 3:** Association Between Anti-HBs Antibody Status, Significant Chronic Diseases, and Healthcare Referrals.

Characteristic		Anti-HBs Antibodies		
		0 (Positive, ≥10 IU/L)	1 (None)	*p*-Value
		n = 859	n = 463	
Significant chronic diseases, n (%) ^χ^				0.042
	Absent (0)	611 (66.78%)	304 (33.22%)	
	Present (1)	247 (61.14%)	157 (38.86%)	
	No data	1 (50.00%)	1 (50.00%)	
Referral to specialist clinic, n (%) ^χ^				0.469
	No (0)	762 (65.30%)	405 (34.70%)	
	Yes (1)	82 (62.12%)	50 (37.88%)	
	No data	15 (65.22%)	8 (34.78%)	
Referral to hospital, n (%) ^χ^				0.847
	No (0)	807 (65.03%)	434 (34.97%)	
	Yes (1)	37 (63.79%)	21 (36.21%)	
	No data	15 (65.22%)	8 (34.78%)	

Legend: ^χ^ Pearson Chi-square test; analyses were performed excluding unavailable data.

**Table 4:** Distribution of children’s vaccination status depending on the presence of major chronic diseases and referrals to specialist clinics and hospitals.

Characteristic		Vaccination Status 0—Complete	Vaccination Status 1—Absent	Vaccination Status 2—Incomplete	No Data	*p*-Value ^χ^
Significant chronic diseases						
	Absent (0)	784 (85.68%)	14 (1.53%)	117 (12.79%)	0 (0.00%)	<0.001
	Present (1)	316 (78.02%)	26 (6.42%)	60 (14.81%)	3 (0.74%)	
	Missing data	0 (0.00%)	0 (0.00%)	2 (100.00%)	0 (0.00%)	
Referral to Specialist Clinic						
	No (0)	981 (84.06%)	31 (2.66%)	153 (13.11%)	2 (0.17%)	0.571
	Yes (1)	106 (80.30%)	5 (3.79%)	20 (15.15%)	1 (0.76%)	
	Missing data	13 (56.52%)	4 (17.39%)	6 (26.09%)	0 (0.00%)	
Referral to Hospital						
	No (0)	1036 (83.48%)	35 (2.82%)	167 (13.46%)	3 (0.24%)	0.680
	Yes (1)	51 (87.93%)	1 (1.72%)	6 (10.34%)	0 (0.00%)	
	Missing data	13 (56.52%)	4 (17.39%)	6 (26.09%)	0 (0.00%)	

Legend: ^χ^ Pearson Chi-square test; analyses were performed excluding unavailable data.

## Data Availability

The datasets used and analyzed during the current study are available from the corresponding author upon reasonable request.
